# Development of Biopolymer Composite Films Using a Microfluidization Technique for Carboxymethylcellulose and Apple Skin Particles

**DOI:** 10.3390/ijms18061278

**Published:** 2017-06-15

**Authors:** Inyoung Choi, Yoonjee Chang, So-Hyang Shin, Eunmi Joo, Hyun Ju Song, Haeyoung Eom, Jaejoon Han

**Affiliations:** 1 Department of Biotechnology, College of Life Sciences and Biotechnology, Korea University, Seoul 02841, Korea; haunlzy@korea.ac.kr (I.C.); bus882@korea.ac.kr (E.J.); hjhs46@korea.ac.kr (H.J.S.); iamhy417@korea.ac.kr (H.E.); 2 Institute of Control Agents for Microorganisms, Korea University, Seoul 02841, Korea; yjchang10@korea.ac.kr; 3 Department of Food Science and Biotechnology, Sungkyunkwan University, Suwon 16419, Korea; sohyang731@naver.com; 4 Department of Food Bioscience and Technology, College of Life Sciences and Biotechnology, Korea University, Seoul 02841, Korea

**Keywords:** apple skin, carboxymethylcellulose, tartaric acid, microfluidization, biodegradable, active packaging film

## Abstract

Biopolymer films based on apple skin powder (ASP) and carboxymethylcellulose (CMC) were developed with the addition of apple skin extract (ASE) and tartaric acid (TA). ASP/CMC composite films were prepared by mixing CMC with ASP solution using a microfluidization technique to reduce particle size. Then, various concentrations of ASE and TA were incorporated into the film solution as an antioxidant and an antimicrobial agent, respectively. Fourier transform infrared (FTIR), optical, mechanical, water barrier, and solubility properties of the developed films were then evaluated to determine the effects of ASE and TA on physicochemical properties. The films were also analyzed for antioxidant effect on 2,2-diphenyl-1-picrylhydrazyl radical scavenging activity and antimicrobial activities against *Listeria monocytogenes*, *Staphylococcus aureus*, *Salmonella enterica*, and *Shigella flexneri*. From the results, the ASP/CMC film containing ASE and TA was revealed to enhance the mechanical, water barrier, and solubility properties. Moreover, it showed the additional antioxidant and antimicrobial properties for application as an active packaging film.

## 1. Introduction

Petrochemical-based plastics such as polyethylene, polypropylene, and polystyrene have been widely used as packaging films due to their availability at low cost and their good mechanical properties. However, the use of plastics has become restricted since they are highly resistant to environmental biodegradation causing many environmental issues [1,2]. As potential replacements for conventional petrochemical plastics, biopolymers such as starch, protein, and lipid, which could be degraded ranging from months to several years, can be applied for the sustainable development of packaging materials since they are non-toxic, biodegradable, and recyclable [3].

The commercial viability of biopolymer-based food packaging is somewhat limited due to the high cost of commercial-scale production using biopolymer materials and inadequate mechanical and barrier properties [4]. Fortunately, it has been reported that a substantial amount of apple skin (roughly 9000 tons) as a biopolymer is left over every year from manufacturing processes [5,6]. Some studies have described the utilization of apple process co-products, called apple skin, as biomass and biodegradable plastic film materials at low cost, which also exhibit antioxidant activity because of the phenolic compounds present in apple skin [4,7]. However, in a previous study, a film made exclusively from apple skin showed poor film-forming ability [8], making it impossible to be applied in the food packaging industry. For these reasons, other materials are clearly needed to be put into apple skin-based products in order to improve the film-forming properties.

Among the various biopolymeric materials, cellulose derivatives are well known for their good film-forming properties and stability [9]. In particular, carboxymethylcellulose (CMC) is a cellulose derivative with carboxymethyl substituents (–CH_2_COOH) bound to some of the hydroxyl groups of the cellulose. CMC can be used as an effective additive to improve product quality and processing properties in various fields, including the food, cosmetic, paper, and textile industries [10]. For the biodegradable film formation, the CMC addition is helpful to improve the film strength and a gas barrier property, probably due to the formation of the film network structure by high polysaccharide and protein contents and the firmly linked chemical bonds, respectively [11]. Especially, Shin et al. [8] demonstrated that the CMC film was better to formulate the film solution with apple skin than other tested polymers, such as gelatin, polylactide, and methylcellulose. Owing to its good film-forming ability, biodegradability, and non-toxicity, CMC has also been applied in edible film formulations [12]. Some studies have also dealt with CMC-based films containing soy protein isolate [13] and silver nanoparticles as an antimicrobial agent [14]. In fact, trials to produce biodegradable films based on apple skin and CMC have already been reported by Lee et al. [15] and Shin et al. [8]. However, the effect of using each material on physicochemical properties has not been determined.

Microfluidization, a high-pressure homogenization technique, is effective at breaking down aggregates and enhancing particle dispersion in a film-forming solution [16]. It can reduce dispersed particle size in a biomass film-forming solution, thus improving miscibility and film properties including appearance, moisture barrier, and mechanical properties [8].

Organic acids with antimicrobial activity are used as preservatives, flavor enhancers, and acidulates in the food industry. These organic acids can be effective agents for ensuring the microbial safety of foods [17]. The antimicrobial activity of organic acids varies depending on the characteristics of each organic acid. Among them, tartaric acid (TA) is a dicarboxylic organic acid present in various fruit-bearing plants and berries, which is affirmed as GRAS (generally recognized as safe, 21CRF184) and can be directly added to food products [18].

Here, we sought (1) to develop apple skin-based bioplastic film formulations and (2) to measure the effect of apple skin extract (ASE), and TA addition to mechanical and water barrier properties as well as the antioxidant and antimicrobial activities of the developed films.

## 2. Results and Discussions

### 2.1. Optical Properties

Optical properties are of great importance in food packaging films since they directly influence consumer acceptance [19]. Overall, the color of films was brown, which was evaluated with color parameters: *L*, *a*, and *b*. The addition of ASE into the apple skin powder (ASP)/CMC composite film affected *L* (lightness), *a* (red to green), and *b* (yellow to blue) parameters (*p* ≤ 0.05) as shown in Table 1. The *L* and *a* values decreased, while the *b* value increased, indicating the film became darker, greener, and yellower. Since the ASE contains a large amount of polyphenol compounds including flavonoids and anthocyanin [20], a change in the ASE content could lead to a change in the color of the biopolymer film, as demonstrated by our previous study [15]. Transparency also gradually decreased with the addition of ASE, for which significant results were obtained (*p* ≤ 0.05).

In the presence of TA, all color variables and transparency of ASP/CMC composite films with ASE and TA changed significantly (*p* ≤ 0.05), as illustrated in Table 1. Compared to the ASP/CMC composite film with 1% ASE, the addition of TA led to decreases in *L* and *b* values as well as transparency, and an increase in *a* value, meaning that the films were highly red and less yellow. The difference in transparency can be ascribed to the contribution of phenolic compounds in ASE and TA to the color of the ASP/CMC composite film [21].

### 2.2. Fourier Transform Infrared (FTIR) Spectroscopy Analysis

**Figure 1:**
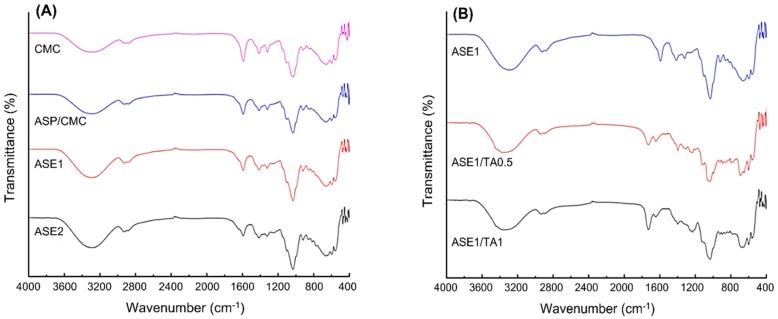
Fourier transform infrared (FTIR) spectra of (**A**) the CMC, ASP/CMC composite film, ASP/CMC film with 1% and 2% ASE, respectively; and (**B**) the ASP/CMC film with 1% ASE and TA (0.5% and 1%, respectively). CMC, carboxymethylcellulose; ASP, apple skin powder; ASE, apple skin extract; TA, tartaric acid; ASP/CMC, ASP/CMC composite film; ASE1, ASP/CMC composite film with 1% ASE; ASE2, ASP/CMC composite film with 2% ASE; ASE1/TA0.5, ASP/CMC composite film with 1% ASE and 0.5% TA; ASE1/TA1, ASP/CMC composite film with 1% ASE and 1% TA.

The spectra of the CMC and ASP/CMC composite film with and without ASE and TA were measured to investigate the interaction between ASP/CMC composite film, ASE, and TA, respectively (Figure 1). The main characteristic bands of CMC are assigned as follows (Figure 1A): The broad band at 3290 cm^−1^ is attributed to stretching of the –OH group and the intense band at 2920 cm^−1^ is due to the C–H stretching vibration. The asymmetric COO– group band can be observed at 1590 cm^−1^. The bands at 1425 and 1325 cm^−1^ are ascribed to –CH_2_ scissoring and –OH group bending vibration, respectively [22]. For ASP/CMC composite films with and without ASE, the main characteristics are almost identical to the CMC spectrum, for the following reasons. On one hand, ASP contains general components of plant cell walls, such as cellulose, lignin, and pectin [23], which shows the similar structure to CMC. On the other hand, ASE does not produce new chemical bonds, but instead might be physically dispersed within the ASP/CMC composite film [8]. The relative intensity, especially at 3290 cm^−1^, is improved as a result of physical interactions and stronger hydrogen bonds with polar groups between mixed polymers [7,24]. The incorporation of ASE into ASP/CMC films can be confirmed by the appearance of the weak C=C stretching band at 1646 cm^−1^, the functional group of phenolic compounds, as the concentration of added ASE increases.

For ASP/CMC with the addition of ASE and TA (Figure 1B), newly developed peaks were as follows. First, band between 3400 and 3300 cm^−1^ was observed due to the –OH group present. The characteristics of C–O and C=O stretching in ester bonds at approximately 1728 and 1230 cm^−1^ were also exhibited by all films containing TA. This resulted from the peak coalescence of ester bonds, which are naturally present in the polysaccharide matrix [25]. In addition, the coalescence peak is a result of the carboxyl groups and ester bond in TA. The overall intensity of the spectra was enhanced with the increased proportion of TA, suggesting esterification and transesterification reactions within the CMC and ASP matrix.

### 2.3. Mechanical Properties

**Figure 2:**
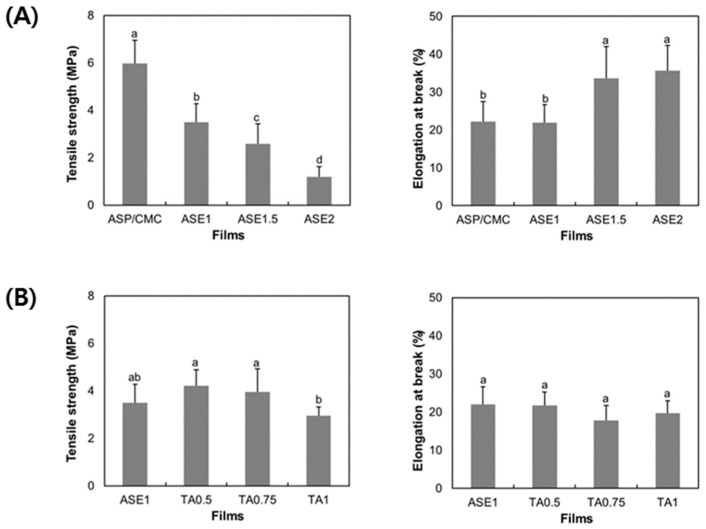
Mechanical properties of (**A**) ASP/CMC composite films supplemented with ASE and (**B**) ASP/CMC composite films supplemented with TA. CMC, carboxymethylcellulose; ASP, apple skin powder; ASE, apple skin extract; TA, tartaric acid; ASP/CMC, ASP/CMC composite film; ASE1, ASP/CMC composite film with 1% ASE; ASE2, ASP/CMC composite film with 2% ASE; TA0.5, ASP/CMC composite film with 1% ASE and 0.5% TA; TA0.75, ASP/CMC composite film with 1% ASE and 0.75% TA; TA1, ASP/CMC composite film with 1% ASE and 1% TA. ^a–d^ Within different concentrations of ASE or TA, different upper-case letters indicate a significant difference (*p* ≤ 0.05).

Mechanical properties (tensile strength and elongation at break) of the films are related to their chemical structures. Tensile strength indicates the maximum stress of films during tensile testing and elongation at break means their potential to stretch. The mechanical properties of ASP/CMC composite films supplemented with TA were presented in Figure 2A. When different amounts of ASE were added, the tensile strength of the films gradually decreased from 5.97 ± 0.97 to 1.19 ± 0.43 MPa, with consistent results among the replicates at each individual concentration (0 to 2%) tested. Regarding the elongation at break of ASP/CMC films with and without ASE, it showed a significant increase (*p* ≤ 0.05) when the concentration of added ASE was over 1.5%. This might have been due to the unbound polyphenols included in ASE, which was physically dispersed in the ASP/CMC matrix. These findings indicate that ASE acted as a plasticizer, inhibiting the formation of intermolecular interactions and promoting hydrogen bonding [26], as indicated by the FTIR spectra. Hager et al. [27] stated that polymer-plasticizer hydrogen bonds can be formed by the polar groups (–OH) of the plasticizer, inducing a decrease in tensile strength. Similar results were reported by Wang et al. [11], who showed that the addition of glycerol to carrot puree films increased their extensibility and reduced their tensile strength. In order to replace the petrochemical-based polymers, these kinds of mechanical properties of biopolymer films should be compared with them. From our previous study [8], the values of elongation at break for both PE and PP were over 400%, which could not be compared with the biopolymer films. Also, the tensile strengths of polyethylene (PE) and polypropylene (PP) were 14.76 MPa and 26.96 MPa, respectively. The results were higher than the ASP/CMC based biopolymer films. However, considering the tensile strength of the only CMC based film (40.51 MPa), we could surely enhance the mechanical property of ASP/CMC in future studies.

Figure 2B shows the mechanical properties of ASP/CMC composite films supplemented with TA. Both tensile strength and elongation at break did not show any tendency to change (*p* > 0.05) at different concentrations of TA. Organic acids, used as antimicrobial and cross-linking agents [16,27], could interact with the polymeric molecules. However, in the present study, TA did not show a cross-linking effect, with no enhancement of the mechanical properties of the films. This might have been due to the production of weak chemical bonds with a small amount of organic acids. The results represent the same trend as that proposed by Hager et al. [27], who stated that low levels (1–2%) of gallic acid had no effect on the mechanical properties of wheat gluten films.

### 2.4. Water Vapor Permeability (WVP)

A major function of a packaging film is to retard moisture transfer between food and the surrounding atmosphere. Water vapor permeability (WVP) can be affected by many factors such as the integrity of films, the hydrophilic–hydrophobic ratio, and the mobility of the polymeric chain [28]. In addition, WVP depends on the number of polar (–OH) groups possessed by a polymer [29].

**Figure 3:**
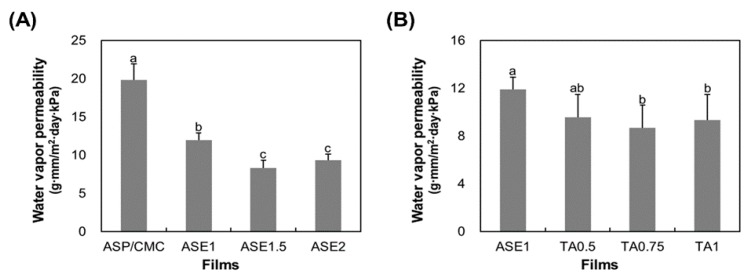
Water vapor permeability for the ASP/CMC films with different concentrations of (**A**) ASE and (**B**) TA. CMC, carboxymethylcellulose; ASP, apple skin powder; ASE, apple skin extract; TA, tartaric acid; ASP/CMC, ASP/CMC composite film; ASE1, ASP/CMC composite film with 1% ASE; ASE2, ASP/CMC composite film with 2% ASE; TA0.5, ASP/CMC composite film with 1% ASE and 0.5% TA; TA0.75, ASP/CMC composite film with 1% ASE and 0.75% TA; TA1, ASP/CMC composite film with 1% ASE and 1% TA. ^a–d^ Within different concentrations of APE, different upper-case letters indicate a significant difference (*p* ≤ 0.05).

The effects of ASE and TA on ASP/CMC composite films are shown in Figure 3. The WVP values for films significantly decreased (*p* ≤ 0.05) from 19.82 ± 2.07 to 8.31 ± 1.00 g·mm/m^2^·day·kPa as different concentrations of ASE were added (Figure 3A). These findings are analogous to the results reported by Du et al. [7] and Rojas-Grau et al. [30], indicating a significant decrease in WVP when the apple skin was added. The lower WVP might be because the ASE containing polyphenolic compounds reduced the “available” unbound polar (–OH) groups in the ASP/CMC polymer matrix through the formation of hydrogen and covalent bonds, while inhibiting interaction between water vapor and the CMC matrix [31].

The WVP results for ASP/CMC films with ASE and TA are shown in Figure 3B. Compared to the film to which only ASE was added, the incorporation of TA led to a reduction of WVP from 11.91 ± 0.99 to 8.69 ± 1.87 g·mm/m^2^·day·kPa. However, statistical analysis did not show a significant (*p* > 0.05) interaction between film components and TA at concentrations under 0.5%. In general, water vapor is transferred through the hydrophilic portion of the film, depending on the hydrophilic-hydrophobic ratio of the film formulation. According to the addition of TA, a hydrophilic hydroxyl group in the ASP/CMC matrix could be substituted with the hydrophobic ester groups, thus decreasing the WVP values of the films [32]. This is consistent with the work of Olivato et al. [25], who investigated the effect of TA on the WVP of thermoplastic starch/polyester blown films. They stated that WVP decreased because a cross-linking reaction reduced the mobility of polymeric chains and made the diffusion of water within the film matrix more difficult when organic acids were used.

### 2.5. Water Solubility

**Figure 4:**
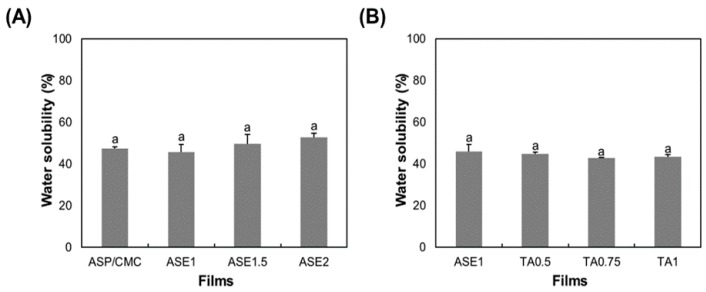
Water solubility of the ASP/CMC composite films, as a function of (**A**) ASE concentrations and (**B**) TA contents. CMC, carboxymethylcellulose; ASP, apple skin powder; ASE, apple skin extract; TA, tartaric acid; ASP/CMC, ASP/CMC composite film; ASE1, ASP/CMC composite film with 1% ASE; ASE2, ASP/CMC composite film with 2% ASE; TA0.5, ASP/CMC composite film with 1% ASE and 0.5% TA; TA0.75, ASP/CMC composite film with 1% ASE and 0.75% TA; TA1, ASP/CMC composite film with 1% ASE and 1% TA. ^a–d^ Within different concentrations of APE, different upper-case letters indicate a significant difference (*p* ≤ 0.05).

The water solubility of the ASP/CMC composite films, as a function of ASE or TA content, is depicted in Figure 4. The water solubility of the ASP/CMC composite (Figure 4A) itself was measured to be 47.41 ± 0.76%, which might have been due to the hydrophilic cellulose structure of ASP and the moisture sorption and water-binding properties of the hydroxyl and carboxyl groups of CMC [33]. The increasing amount of ASE in the ASP/CMC matrix seemed to affect the water solubility of films because of the excessive polyphenols in ASE, but did not show a significant difference (*p* > 0.05). This suggests that the addition of ASE did not interfere with the arrangement of the ASP and CMC matrix since a strong intermolecular reaction did not occur in the ASP/CMC films.

Regarding the addition of TA at various concentrations, no significant difference could be observed (*p* > 0.05), as shown in Figure 4B. Olivato et al. [34] stated that the addition of a large amount of TA can lead to the cross-linking of polymeric molecules, resulting in improved resistance to the dissolution of water in films. However, in this study, the addition of TA did not affect water resistance since lower proportions of TA were added.

### 2.6. Antioxidant Activity of Films

**Figure 5:**
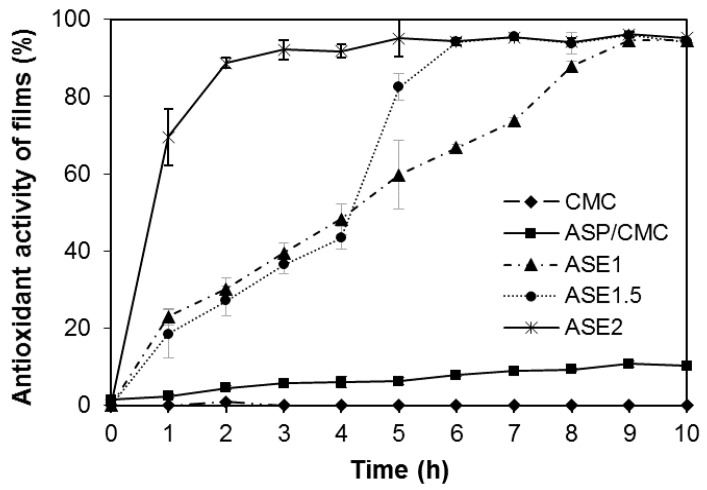
2,2-diphenyl-1-picrylhydrazyl (DPPH) radical scavenging activity of ASP/CMC composite films containing various ASE concentrations. CMC, carboxymethylcellulose; ASP, apple skin powder; ASE, apple skin extract; TA, tartaric acid; ASE1, ASP/CMC composite film containing 1% apple peel extract; ASE1.5, ASP/CMC composite film containing 1.5% apple peel extract; ASE2, ASP/CMC composite film containing 2% apple peel extract.

The antioxidant activity of the films was investigated by measuring the continuous release of antioxidant agents into the liquid medium during storage, as shown in Figure 5. The CMC film did not show any antioxidant activity during storage, since antioxidant compounds were not present in the polymer. On the other hand, our previous study showed that the apple skin contains 24.94 mg gallic acid equivalents/g dried apple skin of total phenolic compounds and 8.85 mg catechin equivalents/g dried apple skin, significantly higher than other herb and fruit contents [15]. Among the components of apple skin, ASP, made from the remnant apple skin after ASE extraction, could cause a slight increase in antioxidant activity of the ASP/CMC composite film since the main components of ASP were cellulose, pectin, and lignin. But, the addition of ASE into the ASP/CMC composite film markedly improved the antioxidant activity of the films in a concentration-dependent manner, as the ASE, which was soluble phenolic compounds, included antioxidant agents such as chlorogenic acid, hydroxycinnamic acid, procyanidin B3, rutin, and flavonols extracted from apple skin [35]. Thus, the antioxidant activity of films with ASE varied depending on the ASE concentration. Specifically, the ASP/CMC film with 2% ASE showed the highest antioxidant activity, and at the same time, the film rapidly reached the top of the value after 3 h, as compared to the other films. Since ASE, physically dispersed in the ASP/CMC matrix, might exist as an unbound material if present in excess, it can easily be released from the matrix and result in antioxidant activity. These findings are similar to those reported by Peng et al. [36].

### 2.7. Antimicrobial Activity of Films

The antimicrobial activities of ASP/CMC composite films containing 1% ASE and different concentrations of TA (0.75% and 1%) was investigated for four bacteria: two gram-positive bacteria (*Listeria monocytogenes* and *Staphylococcus aureus*) and two gram-negative bacteria (*Salmonella enterica* and *Shigella flexneri*). Since the single components, CMC and ASE, did not show antimicrobial activity against these bacteria in our previous study, the antimicrobial testing of these materials was not undertaken.

**Figure 6:**
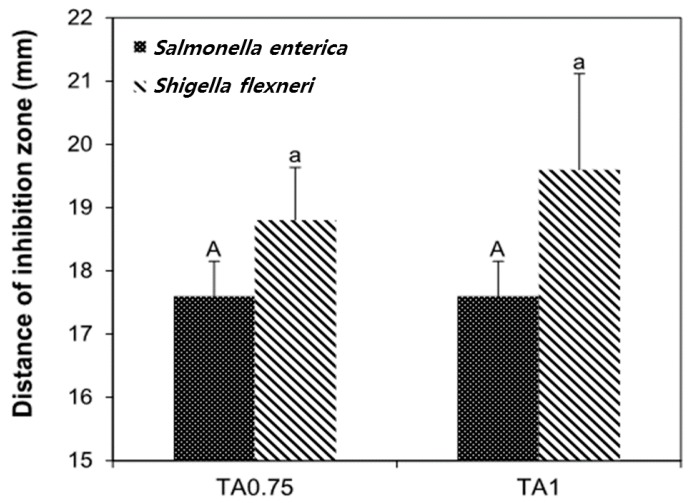
Distance of inhibition zone formed by the ASP/CMC containing 1% ASE and different concentrations of TA (0.75% and 1.0%) against *Salmonella enterica* and *Shigella flexneri*. CMC, carboxymethylcellulose; ASP, apple skin powder; ASE, apple skin extract; TA, tartaric acid. TA0.75, ASP/CMC composite film containing 1% APE and 0.75% TA; TA1, ASP/CMC composite film containing 1% APE and 1% TA. ^A^ Within different concentrations of TA against *S. flexneri*, different upper-case letters indicate a significant difference (*p* ≤ 0.05); ^a^ Within different concentrations of TA against *S. enterica*, different lower-case letters for each TA concentration indicate a significant difference (*p* ≤ 0.05).

The ASP/CMC containing both ASE and TA exhibited an inhibitory effect toward gram-negative bacteria (Figure 6), but not gram-positive bacteria (data not shown). Although gram-positive bacteria are generally more sensitive to organic acids than gram-negative ones [37], some gram-positive bacteria can survive under acidic conditions due to their acid tolerance, similar to these findings. In contrast, the growth inhibitions of gram-negative bacteria such as *S. enterica* and *S. flexneri* were observed in the ASP/CMC film with ASE and TA. The inhibitory effect of an organic acid is ascribed to the impact of pH reduction in the cytoplasm. The non-dissociated organic acids, penetrating the lipid membrane of bacterial cells, can be dissociated into hydrogen ions and anions, followed by a pH reduction in the interior of microbial cells. This can alter the permeability of the cell membrane, disrupt substrate transport, and reduce the proton motive force, resulting in the inhibition of bacterial growth [38]. Thus, ASP/CMC film with TA could show the inhibitory effect on *S. enterica* and *S. flexneri*, with no significant statistical analysis on the added concentration of TA (*p* > 0.05).

## 3. Materials and Methods

### 3.1. Materials

CMC was purchased from Sigma-Aldrich Co., Ltd. (St. Louis, MO, USA) and glycerol was purchased from Daejung Chemicals and Metals Co. (Siheung, Korea). *L*-(+)-TA was provided by Junsei Chemical Co. (Tokyo, Japan).

### 3.2. Preparation of ASP and ASE

Fresh apples were obtained from Nonghyup local market (Yungjoo, Korea). Apple skin (average thickness of 0.36 ± 0.10 mm) was obtained by washing and peeling fresh apples. The obtained apple skin was then dried in an oven (VS-1202D2; Vision Scientific Co., Daejeon, Korea) at 45 °C for 24 h. After drying, the apple skin was finely pulverized with an electric grinder (Daesung Artron Co., Seoul, Korea) and stored at −20 °C until extraction. To prepare ASE, the pulverized ASP was mixed in a solvent of 90% ethanol at a ratio of 1:10 (*w*/*v*) under vigorous whisking (Lab Stirrer MS-280; Misung Co., Ltd., Seoul, Korea) and sonicated at 25 °C for 30 min. After extraction, the extract solution was filtered twice through Whatman No. 2 filter paper (Whatman Inc., Clifton, NJ, USA). The obtained filtrate was then concentrated using a rotary evaporator (Rotavapor RE121; Buchi, Fawil, Switzerland) to evaporate the extract solvent, 90% ethanol, and then frozen at −40 °C and freeze-dried (Heto FD3; Heto-Holten, Allerod, Denmark). The dried ASE sample was weighed to calculate the soluble content yield. The sample was then stored in an air-tight container at −20 °C until further analysis. The ASP remaining on the filter paper was desiccated in a drying oven at 45 °C for 24 h. It was then ground more finely in an electric grinder and passed through a standard sieve (U.S. No. 100) to obtain only particles smaller than 149 µm. The separated ASP was collected and used to develop ASP/CMC composite films.

### 3.3. Film Preparation

The concentration of components in each ASP/CMC composite film-forming solution is shown in Table 2. All tests were carried out at room temperature (25 °C), without heating. The CMC film solution was prepared by dissolving 1.5 g of CMC and 0.5 g of glycerol as a plasticizer in 50 mL of distilled water for 24 h with stirring. The apple skin film solution can be obtained by mixing 1.5 g of ASP and 0.5 g of glycerol together in 50 mL of distilled water. This ASP mixture was introduced into a microfluidization processor (M-110S; Microfluidics 137 International Co., Newton, MA, USA), which had an H10Z (100 µm) interaction 138 chamber, operated at 150 MPa for three passes. The ASP/CMC composite film-forming solution was prepared by mixing the same mass ratio (*v*/*v*) of CMC solution and microfluidized ASP solution. After the preparation of ASP/CMC film solution, ASE was added as an antioxidant at final concentrations of 1%, 1.5%, and 2%. TA, an antimicrobial agent, was added to the ASP/CMC film solution with 1% ASE at final concentrations of 0.5%, 0.75%, and 1%. The final solution was homogenized at 18,000× *g* for 1 min (SR30; Mtop-Korea, Seoul, Korea). After homogenization, the final solution was degassed using an aspirator to remove air bubbles. Each prepared film-forming solution was evenly poured onto a Petri dish. The casting plates were then placed and dried in an incubator at room temperature (22 ± 3 °C) for at least 24 h.

### 3.4. Film Conditioning and Thickness

The dried films were peeled off from the dishes and stored in a thermo-hygrostat at 25 °C and 50% relative humidity (RH) (Lab-Made 011; Sejong Scientific Co., Bucheon, Korea) for 48 h or more before testing. The film thickness was measured using a digital micrometer (ID-C112X; Mitutoyo Co., Kawasaki, Japan), with an accuracy of 0.1 µm. It was used for assessing the mechanical and barrier properties of the films.

### 3.5. Optical Properties

The optical properties of the films were measured using a CR-400 colorimeter (Konica Minolta, Osaka, Japan), which was calibrated against a standard white background. Film specimens were placed on the surface of a standard white plate, and the Hunter parameters *L* (lightness; 100 = white, 0 = black), *a* (redness; positive = redness, negative = greenness), and *b* (yellowness; positive = yellowness, negative = blueness) color values were used. The total color difference (Δ*E*) was calculated as follows:

(1) $$\Delta E=\sqrt{{\left(L-L_{0}\right)}^{2}+{\left(a-a_{0}\right)}^{2}+{\left(b-b_{0}\right)}^{2}}$$

where *L*_0_, *a*_0_, and *b*_0_ are the Hunter color values of the standard white plate (*L*_0_ = 96.37, *a*_0_ = 0.19, and *b*_0_ = 1.68). Measurements were taken as the average of those at five locations on each sample. Transparency (T_660_) of the films was determined with a UV-Vis spectrophotometer (UV mini-1640; Shimadzu, Kyoto, Japan) at 660 nm in the transmittance mode. The film sample was cut into a strip (25.4 mm × 50 mm) and directly placed into a film holder. The optical properties of each type of film were measured from five replicates.

### 3.6. FTIR Spectroscopy

IR spectra of the films were obtained using a Varian 640-IR (Varian, Inc., Palo Alto, CA, USA) fitted with attenuated total reflection (ATR) accessories. FTIR spectroscopy was used to observe the formation of chemical bonds among the film-forming polymer materials, ASE and TA. The FTIR spectrum of each film was measured in the transmittance mode in the wavenumber range between 4000 and 400 cm^−1^, and recorded with a total of 32 scans at 4 cm^−1^ resolution.

### 3.7. Mechanical Properties

Tensile strength and elongation at break were determined using a texture analyzer (TAXT Plus 50; Stable Micro Systems Ltd., Surrey, UK) in accordance with the ASTM standard method D882-91 (1995). Prior to the measurement, the films were conditioned in a thermo-hygrostat at 25 °C and 50% RH for 48 h. Each film was cut into a strip (25.4 mm × 100 mm), which was placed between the grip heads of the machine. The initial grip separation rate was 50 mm/s, and operation was performed at a cross-head speed of 0.5 mm/s. At least 10 replicates were analyzed for each film type.

### 3.8. Water Vapor Permeability

WVP was tested gravimetrically using the cup method described by Han et al. [39] with slight modifications. Each film sample was placed on a cylindrical cup containing anhydrous calcium chloride and covered with a ring-shaped lid. The hermetically sealed cups were pre-weighed using electronic scales with a precision of 0.001 g and kept in a thermo-hygrostat adjusted to 25 °C and 50% RH. After 24 h, the cups were weighed and WVP was calculated as follows:

WVP (g∙mm/m^2^∙day∙kPa) = (W·x)/(A·t·(P_2_ − P_1_)) (2)

where W is the difference in weight of the cup after 24 h (g), x is the average film thickness (mm), A is the area of the film exposed to air (m^2^), t is the storage time (1 day), and P_2_ − P_1_ is the difference in vapor across the film (kPa). The WVP of each film was determined from five replicates.

### 3.9. Water Solubility

Film samples (25 mm × 50 mm) were dried in a drying oven at 100 °C for 24 h, and then weighed to measure the initial water content of the film. The dried samples were immersed in 30 mL of distilled water for 24 h at 25 °C in an incubator. After the remnant water had been discarded, the samples were placed in a dry oven at 100 °C for 24 h, and then, the final weight of the solid contents was measured. Water solubility of the films was calculated according to the following formula:

Water solubility (%) = (W_0_ − W_f_)/W_0_ × 100 (3)

where W_0_ is weight of the initial dried film and W_f_ is the weight of the insoluble dried film. The water solubility of each film was determined from five replicates.

### 3.10. Antioxidant Activity of Films

The antioxidant activity of the films was determined using the 2,2-diphenyl-1-picrylhydrazyl (DPPH) radical scavenging assay, based on the method of Park et al. [40]. The films were cut into 23 mm × 40 mm pieces and adhered to the inner wall of a glass vial, containing 2.7 mL of 0.1 mM DPPH solution. The vials were stored in the dark for 10 h and the absorbance of the supernatant was measured at 517 nm every 1 h. Vials without films and with only a CMC film were prepared as blanks and negative controls, respectively. The antioxidant activity of the films was calculated as follows:

Antioxidant activity (%) = (Abs_0_ − Abs_s_)/Abs_0_ × 100 (4)

where Abs_0_ is the absorbance of the blank and Abs_s_ is the absorbance of the film sample. All measurements were replicated three times.

### 3.11. Antimicrobial Activity of Films

An agar disc diffusion assay was carried out to investigate the antimicrobial activity of the APE films against four bacterial strains: *L. monocytogenes*, *S. aureus*, *S. enterica*, and *S. flexneri*. Each bacterial culture was transferred to 10 mL of Tryptic Soy Broth (TSB; BBL/Difco, Sparks, MD, USA) using a 10-µL sterile inoculation loop and incubated at 37 °C three times with 24 h intervals. A 1-mL bacterial suspension containing 10^5^ CFU/mL bacteria was inoculated in Tryptic Soy Agar (TSA; BD/Difco). Films cut into a disc of 15 mm in diameter were placed on the agar, and then, the plates were incubated at 37 °C for 24 h. The diameter of the inhibitory zones (mm) surrounding the film discs were then measured in triplicate.

### 3.12. Statistical Analysis

Statistical Analysis System (SAS) software, version 9.3 (SAS Institute, Cary, NC, USA), was used to express the mean ± standard deviation of the experimental results. The General Linear Models Procedure was used for analysis of variance, with the significance of differences between the main-effect means being analyzed by the Student-Newman-Keuls test. Statistical significance was identified at the 95% confidence level (*p* ≤ 0.05).

## 4. Conclusions

Apple skin was successfully mixed with CMC to develop an eco-friendly biopolymer film. When ASE and TA were incorporated into the ASP and CMC composite film, the WVP and tensile strength decreased and the elongation at break increased. Moreover, the antioxidant activities of the films were significantly enhanced depending on the ASE concentration, and antimicrobial effects of the films against *S. enterica* and *S. flexneri* were observed due to the TA addition. The present study shows the way to enhance the mechanical properties of films based on apple skin and CMC with the functionality enhancement, but it needs further studies, such as an application test, in order to be applied in the food packaging industry. However, we believe that the developed biopolymer film has the potential to be used in some following areas; fresh food packaging to inhibit microbial growth on the food surface due to the antimicrobial activity, high oil content food packaging to minimize lipid oxidation due to the antioxidant activity, and light sensitive food packaging to reduce the acceleration of food rancidity due to the brownish color of the biopolymer film. Therefore, it can partially show a promising alternative to synthetic polymers, which can be utilized as a non-toxic, antimicrobial, antioxidant film, and a light barrier film due to the brown color.

## Figures and Tables

**Table 1:** Optical properties of apple skin powder/carboxymethylcellulose (ASP/CMC) composite films incorporated with different concentrations of apple skin extract (ASE) and tartaric acid (TA).

Color Parameters	Bioplastic Film						
	ASP/CMC	ASE1	ASE1.5	ASE2	ASE1/TA0.5	ASE1/TA0.75	ASE1/TA1
*L*	92.85 ± 0.31 ^A^	89.43 ± 0.32 ^B,d^	88.34 ± 0.18 ^C^	88.46 ± 0.21 ^C^	89.39 ± 0.49 ^c^	88.51 ± 0.20 ^b^	85.30 ± 0.22 ^a^
*a*	1.28 ± 0.06 ^A^	0.31 ± 0.09 ^B,d^	0.43 ± 0.07 ^B^	0.32 ± 0.10 ^B^	5.58 ± 0.30 ^c^	8.20 ± 0.26 ^b^	11.48 ± 0.27 ^a^
*b*	12.78 ± 0.22 ^C^	26.95 ± 0.78 ^B,a^	30.99 ± 0.40 ^A^	31.47 ± 0.38 ^A^	19.21 ± 0.49 ^b^	18.25 ± 0.27 ^c^	22.33 ± 0.30 ^d^
Transparency (%)	49.20 ± 2.45 ^A^	37.60 ± 0.90 ^B,a^	31.54 ± 0.89 ^C^	27.38 ± 0.53 ^D^	21.06 ± 0.44 ^b^	20.48 ± 0.79 ^b^	20.16 ± 1.37 ^b^

^A–C^ Within different concentrations of ASE, different upper-case letters indicate a significant difference (*p* ≤ 0.05); ^a–d^ Within different concentrations of TA, different lower-case letters indicate a significant difference (*p* ≤ 0.05). CMC, carboxymethylcellulose; ASP, apple skin powder; ASE, apple skin extract; TA, tartaric acid. ASP/CMC, ASP/CMC composite film without ASE; ASE1, ASP/CMC composite film with 1% ASE; ASE1.5, ASP/CMC composite film with 1.5% ASE; ASE2, ASP/CMC composite film with 2% ASE; ASE1/TA0.5, ASP/CMC composite film with 1% ASE and 0.5% TA; ASE1/TA0.75, ASP/CMC composite film with 1% ASE and 0.75% TA; ASE1/TA1, ASP/CMC composite film with 1% ASE and 1% TA.

**Table 2:** Composition of bioplastic film-forming solutions.

Bioplastic Film	Weight (g) of Dry Matter in Distilled Water (100 mL)				
	CMC	ASP	Glycerol	ASE	TA
CMC	3	-	1	-	-
ASP/CMC	1.5	1.5	1	-	-
ASE1	1.5	1.5	1	1	-
ASE1.5	1.5	1.5	1	1.5	-
ASE2	1.5	1.5	1	2	-
ASE1/TA0.5	1.5	1.5	1	1	0.5
ASE1/TA0.75	1.5	1.5	1	1	0.75
ASE1/TA1	1.5	1.5	1	1	1

CMC, carboxymethylcellulose; ASP/CMC, ASP/CMC composite film; ASE1, ASP/CMC composite film with 1% ASE; ASE1.5, ASP/CMC composite film with 1.5% ASE; ASE2, ASP/CMC composite film with 2% ASE; ASE1/TA0.5, ASP/CMC composite film with 1% ASE and 0.5% TA; ASE1/TA0.75, ASP/CMC composite film with 1% ASE and 0.75% TA; ASE1/TA1, ASP/CMC composite film with 1% ASE and 1% TA. CMC, carboxymethylcellulose; ASP, apple skin powder; ASE, apple skin extract; TA, tartaric acid.

## Abbreviations

CMC	carboxymethylcellulose
TA	tartaric acid
ASE	apple skin extract
ASP	apple skin powder
WVP	water vapor permeability
